# Effectiveness of DialBetesPlus, a self-management support system for diabetic kidney disease: Randomized controlled trial

**DOI:** 10.1038/s41746-024-01114-8

**Published:** 2024-04-27

**Authors:** Kayo Waki, Mitsuhiko Nara, Syunpei Enomoto, Makiko Mieno, Eiichiro Kanda, Akiko Sankoda, Yuki Kawai, Kana Miyake, Hiromichi Wakui, Yuya Tsurutani, Nobuhito Hirawa, Tadashi Yamakawa, Shiro Komiya, Akihiro Isogawa, Shinobu Satoh, Taichi Minami, Tamio Iwamoto, Tatsuro Takano, Yasuo Terauchi, Kouichi Tamura, Toshimasa Yamauchi, Masaomi Nangaku, Naoki Kashihara, Kazuhiko Ohe

**Affiliations:** 1 Department of Biomedical Informatics, Graduate School of Medicine, The University of Tokyo, Tokyo, Japan; 2 Department of Planning, Information and Management, University of Tokyo Hospital, Tokyo, Japan; 3 Department of Diabetes and Metabolic Diseases, Graduate School of Medicine, The University of Tokyo, Tokyo, Japan; 4 Department of Metabolism and Endocrinology, Akita University Graduate School of Medicine, Akita, Japan; 5 Department of Medical Informatics, Center for Information, Jichi Medical University, Shimotsuke, Japan; 6 Medical Science, Kawasaki Medical School, Kurashiki, Japan; 7 Department of Medical Science and Cardiorenal Medicine, Yokohama City University Graduate School of Medicine, Yokohama, Japan; 8 Endocrinology and Diabetes Center, Yokohama Rosai Hospital, Yokohama, Japan; 9 Department of Nephrology and Hypertension, Yokohama City University Medical Center, Yokohama, Japan; 10 Department of Endocrinology and Diabetes, Yokohama City University Medical Center, Yokohama, Japan; 11 Division of Diabetes, Mitsui Memorial Hospital, Tokyo, Japan; 12 Department of Endocrinology and Metabolism, Chigasaki Municipal Hospital, Chigasaki, Japan; 13 Department of Diabetes and Endocrinology, Saiseikai Yokohamashi Nanbu Hospital, Yokohama, Japan; 14 Department of Nephrology and Hypertension, Saiseikai Yokohamashi Nanbu Hospital, Yokohama, Japan; 15 Department of Diabetes and Endocrinology, Fujisawa City Hospital, Fujisawa, Japan; 16 Department of Endocrinology and Metabolism, Yokohama City University Graduate School of Medicine, Yokohama, Japan; 17 Division of Nephrology and Endocrinology, Graduate School of Medicine, The University of Tokyo, Tokyo, Japan; 18 Department of Nephrology and Hypertension, Kawasaki Medical School, Kurashiki, Japan

**Keywords:** Type 2 diabetes, Lifestyle modification, Kidney diseases

## Abstract

We evaluated the effectiveness of a mobile health (mHealth) intervention for diabetic kidney disease patients by conducting a 12-month randomized controlled trial among 126 type 2 diabetes mellitus patients with moderately increased albuminuria (urinary albumin-to-creatinine ratio (UACR): 30-299 mg/g creatinine) recruited from eight clinical sites in Japan. Using a Theory of Planned Behavior (TPB) behavior change theory framework, the intervention provides patients detailed information in order to improve patient control over exercise and dietary behaviors. In addition to standard care, the intervention group received DialBetesPlus, a self-management support system allowing patients to monitor exercise, blood glucose, diet, blood pressure, and body weight via a smartphone application. The primary outcome, change in UACR after 12 months (used as a surrogate measure of renal function), was 28.8% better than the control group’s change (*P* = 0.029). Secondary outcomes also improved in the intervention group, including a 0.32-point better change in HbA1c percentage (*P* = 0.041). These improvements persisted when models were adjusted to account for the impacts of coadministration of drugs targeting albuminuria (GLP-1 receptor agonists, SGLT-2 inhibitors, ACE inhibitors, and ARBs) (UACR: −32.3% [95% CI: −49.2%, −9.8%] between-group difference in change, *P* = 0.008). Exploratory multivariate regression analysis suggests that the improvements were primarily due to levels of exercise. This is the first trial to show that a lifestyle intervention via mHealth achieved a clinically-significant improvement in moderately increased albuminuria.

## Introduction

Globally, 537 million people aged 20–79 suffer from diabetes^1^. Diabetic kidney disease (DKD), one of the most common and costly complications of diabetes^2^, is a leading cause of kidney failure and significantly increases cardiovascular disease^3,4^. Urinary albumin-to-creatinine ratio (UACR) is an independent factor in predicting kidney prognosis, diabetic retinopathy, and macrovascular disorders. Reduction of albuminuria is associated with decreased risk of death and kidney failure^5–7^, with a 30% one-year reduction associated with improved cardiovascular and kidney outcomes^8^. UACR correlates well with renal outcome and is an important surrogate endpoint in clinical trials^5,6,9^.

Moderately increased albuminuria is associated with cardiovascular events, and controlling it requires a multifactorial approach. Mobile Health (mHealth, the use of mobile phones and other connected devices to improve health) can provide such an approach. Although pharmacological interventions such as renin-angiotensin-aldosterone system (RAAS) inhibitors, glucagon-like peptide (GLP)-1 receptor agonists, and sodium-glucose cotransporter (SGLT)-2 inhibitors can slow DKD’s progression, polypharmacy and adverse drug events pose clinical challenges^10^. Lifestyle modifications for diet and physical activity along with improved management of glucose, blood pressure (BP), and lipids are a cost-effective therapeutic adjunct to pharmacological treatment^11–14^. Improvements in HbA1c are associated with improved renal outcomes^15,16^. There is evidence that lifestyle modifications can improve kidney function and postpone the progression of DKD^17–19^, though the mechanisms are unclear and research in the area is ongoing. Interventions using mHealth to support patient lifestyle self-management have been shown to control blood glucose levels in type 2 diabetes (T2D) patients^20,21^ and improve physical activity, diet, and medication adherence^22^, but have not previously been shown to improve kidney function. Our mHealth intervention, DialBetesPlus, is an improved version of DialBetics, a self-management support system for patients with T2D that improved HbA1c by 0.4% in a three month 54-patient randomized controlled trial (RCT)^23^. DialBetesPlus helps patients monitor exercise, blood glucose, diet, blood pressure, and body weight via a smartphone application.

This study adds DialBetesPlus to usual care among patients with early-stage DKD to assess its impact, relative to a control group receiving usual care, on albuminuria, kidney function, glycemic control, BP, lipid profile, body mass index (BMI), quality of life (QOL), and diabetes self-management.

## Results

**Fig. 1:**
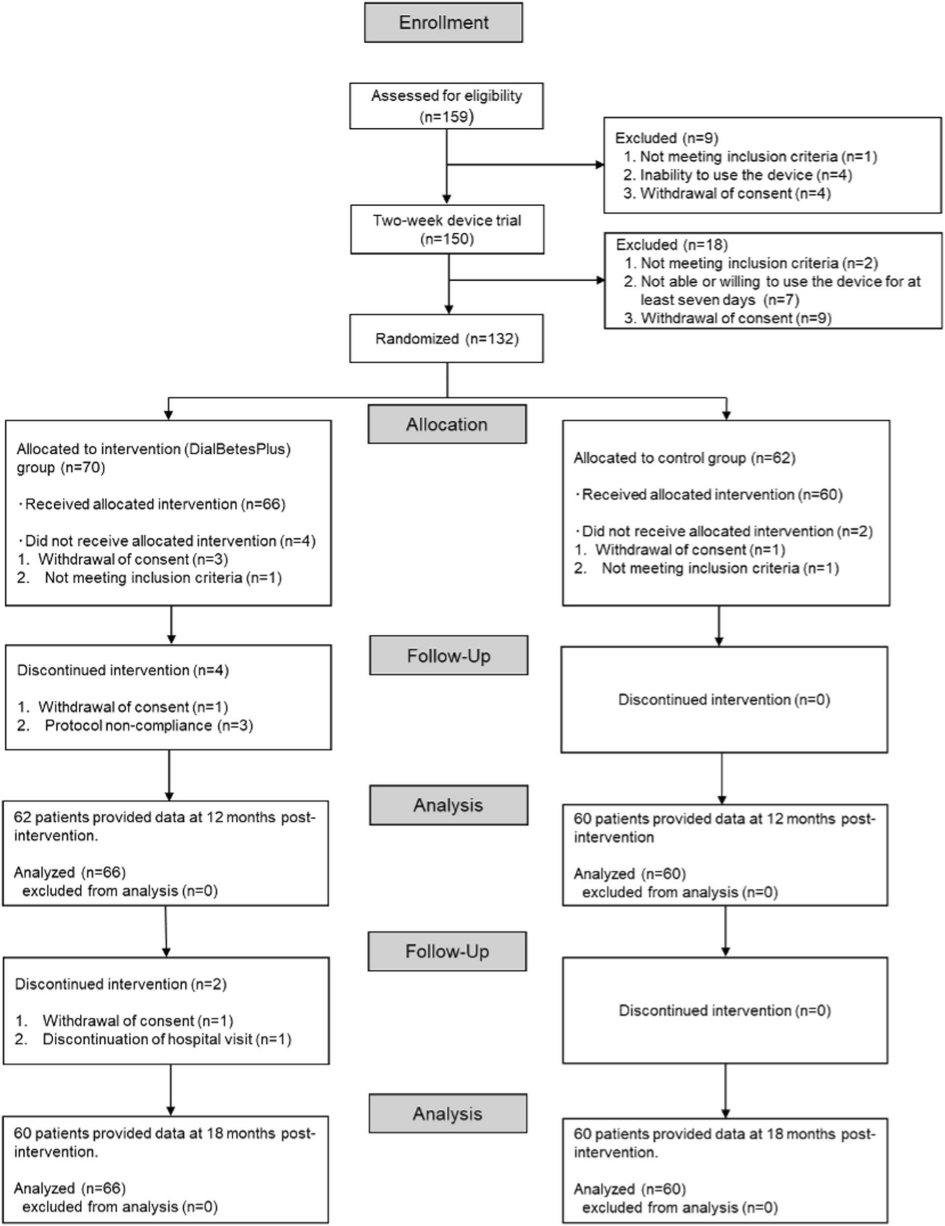
CONSORT flow diagram. After randomization and pre-intervention dropouts, 66 intervention and 60 control patients began the intervention. 62 and 60 remained at 12 months, and 60 and 60 remained at 18 months.

We started recruiting patients on July 1, 2018, and we completed recruitment on August 31, 2019, after enrolling 159 patients. We ended the study on April 6, 2021 upon completion of the follow-up at week 72 for the final patient. 27 of 159 subjects were ineligible—nine identified as such before the two week device trial, and 18 identified after the trial (two newly recognized as not meeting inclusion criteria, seven not able or willing to use the device for at least seven days, and nine withdrawing consent for various personal reasons). The remaining 132 subjects were randomly assigned between groups, yielding 66 (intervention) and 60 (control) at baseline and 62 (intervention) and 60 (control) at 12 months, with 59 (control) having paired baseline and 12-month UACR and HbA1c values (Fig. 1). All 66 intervention and 60 control group patients were included in our Full Analysis Set results, including three intervention patients who were non-compliant to the protocol. Baseline characteristics were well matched (Table 1), although a significantly higher proportion in the intervention group were taking the GLP-1 receptor agonists (37.9% versus 15.0% in the control group, *P* = 0.005).

**Table 1:** Baseline characteristics of participants (*n* = 126)

Characteristics	Control (*n* = 60)		Intervention (*n* = 66)		*P*	Comments
Age (years)	60.3	±8.7	58.6	±10.1	0.312	
Sex					0.546	
Male	46	(76.7)	47	(71.2)		
Female	14	(23.3)	19	(28.8)		
Physical parameters						
BMI (kg/m^2^)	28.3	±4.0	28.7	±5.3	0.696	Obese
Systolic BP (mmHg)	133.1	±16.5	133.1	±16.4	0.990	Slightly higher than 130 goal
Diastolic BP (mmHg)	83.0	±10.2	81.0	±11.3	0.305	Slightly higher than 80 goal
Smoking status, n (%)					0.684	
Non-smoker	23	(38.3)	30	(45.5)		
Current smoker	13	(21.7)	14	(21.2)		
Ex-smoker	24	(40.0)	22	(33.3)		
Duration of diabetes (years)	12.6	±6.4	14.0	±7.9	0.274	
Laboratory test						
FPG (mg/dL)	138.5	(124.0 to 159.5)	150.5	(123.0 to 185.5)	0.354	
HbA1c (%)	7.5	(6.9 to 7.9)	7.5	(7.1 to 8.2)	0.284	Higher than 7% goal
LDL cholesterol (mg/dL)	104.5	(77.0 to 117.5)	97.5	(78.0 to 112.0)	0.422	Met 120 mg/L goal
HDL cholesterol (mg/dL)	49.0	(44.0 to 60.7)	48.3	(41.7 to 60.0)	0.528	Met 40 mg/dL goal
Triglycerides (mg/dl)	152.5	(101.5 to 262.5)	161.5	(100.0 to 268.0)	0.575	Higher than 150 mg/dL goal
Creatinine (mg/dL)	0.8	(0.7 to 0.9)	0.8	(0.6 to 0.9)	0.348	
eGFR (mL/min/1.73m^2^)	71.2	(62.8 to 84.0)	76.4	(59.5 to 85.7)	0.307	
UACR (mg/gCr) ^a^	30.1	±2.8	36.7	±2.9	0.297	
Anti-diabetic medications						
Metformin	43	(71.7)	48	(72.7)	1.000	
Thiazolidinedione	6	(10.0)	12	(18.2)	0.213	
Sulfonylurea	13	(21.7)	10	(15.2)	0.366	
Glinide	6	(10.0)	4	(6.1)	0.517	
α-GI	11	(18.3)	17	(25.8)	0.392	
DPP-4 inhibitor	35	(58.3)	27	(40.9)	0.074	
SGLT-2 inhibitor	34	(56.7)	40	(60.6)	0.718	
GLP-1 receptor agonist	9	(15.0)	25	(37.9)	0.005	Higher in the intervention group.
Insulin	23	(38.3)	27	(40.9)	0.856	
Lipid-lowering medications	46	(76.7)	48	(72.7)	0.684	
Statins	40	(66.7)	43	(65.2)	1.000	
Ezetimibe	6	(10.0)	9	(13.6)	0.591	
Fibrates	5	(8.3)	4	(6.1)	0.735	
Other	6	(10.0)	12	(18.2)	0.213	
Anti- hypertensive medications	38	(63.3)	50	(75.8)	0.174	
ACE inhibitor	2	(3.3)	9	(13.6)	0.057	No statistically significant difference, but higher in the intervention group.
ARB	36	(60.0)	36	(54.5)	0.591	
Calcium channel blocker	26	(43.3)	40	(60.6)	0.074	
b-Blocker	3	(5.0)	1	(1.5)	0.346	
αβ-Blocker	3	(5.0)	8	(12.1)	0.212	
α1-Blocker	1	(1.7)	4	(6.1)	0.368	
Diuretics	8	(13.3)	12	(18.2)	0.477	
Comorbidities						
Coronary Artery Disease	6	(10.0)	11	(16.7)	0.308	
Cerebrovascular Disease	3	(5.0)	8	(12.1)	0.212	
Peripheral Artery Disease	1	(1.7)	2	(3.0)	1.000	
Diabetic retinopathy	10	(16.7)	9	(13.6)	0.804	
Diabetic neuropathy	12	(20.0)	19	(28.8)	0.303	
ADDQoL score	−1.6	±1.7	−1.7	±1.5	0.704	
SDSCA score						
Diet	16.1	±6.6	17.3	±6.8	0.345	
Exercise	6.2	±4.3	5.5	±3.7	0.339	
Self-monitoring of blood glucose	5.5	±6.3	5.3	±6.0	0.924	
Foot care	18.5	±6.7	18.6	±7.5	0.914	

^a^UACR is expressed as geometric mean.

Data are expressed as mean ± SD or median (25^th^ to 75^th^ percentile) or frequency (%). Comparisons between groups use t test or Wilcoxon test for continuous variables and Fisher exact test or Chi square test for categorical variables.

### Effect of intervention on behavior

**Fig. 2:**
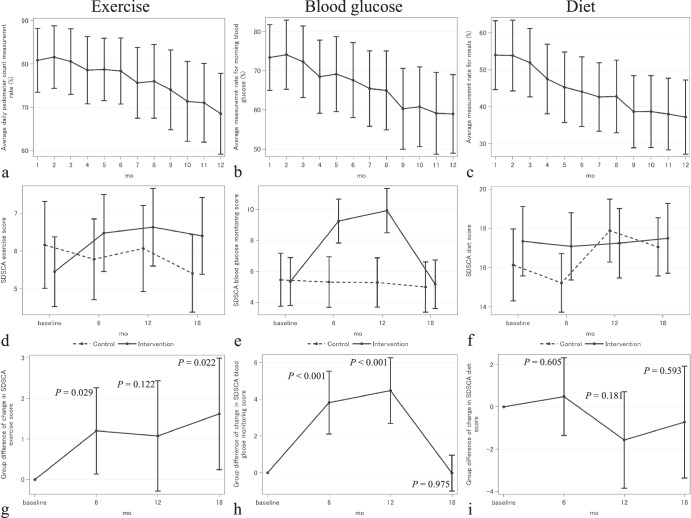
Effect of intervention on behavior. **a** measurement rate of step count **b** measurement rate of blood glucose **c** measurement rate of meals **d** SDSCA score for exercise **e** SDSCA score for blood glucose monitoring **f** SDSCA score for diet **g** difference between intervention and control group of change in SDSCA score for exercise **h** difference between intervention and control group of change in SDSCA score for blood glucose monitoring i) difference between intervention and control group of change in SDSCA score for diet. Error bars represent 95% confidence interval.

**Fig. 3:**
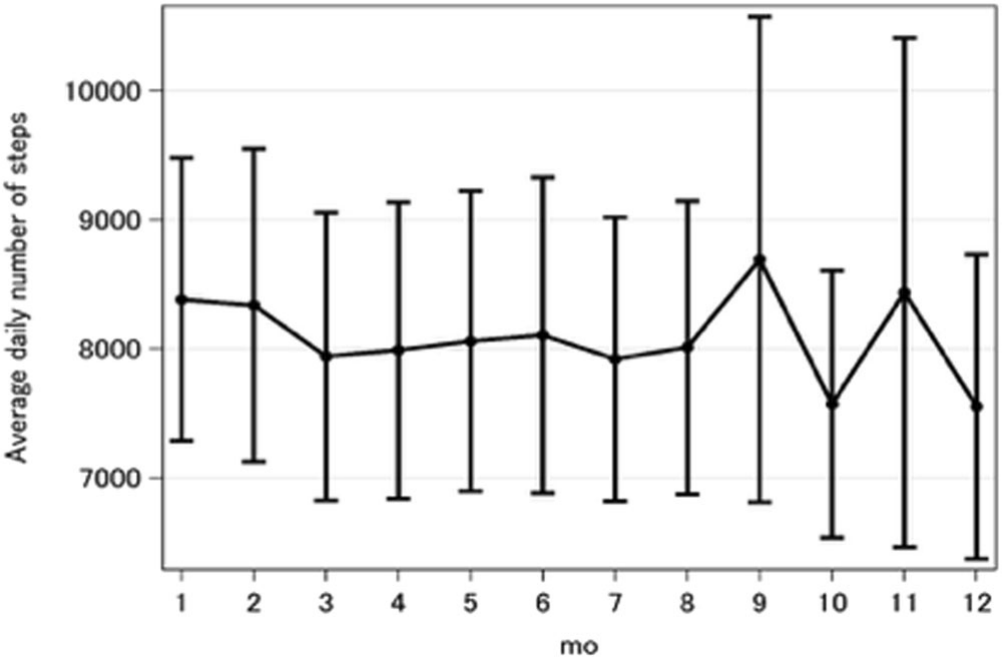
Effect of intervention on daily step count. The daily number of steps were averaged over each month of the intervention and all patients in the intervention group, after conservatively eliminating days indicating lack of use of the pedometer^61^ (days with fewer than 100 steps recorded, representing 23.7% of patient days.). Error bars represent 95% confidence interval.

The intervention uses monitoring and feedback with a goal of increasing exercise behavior. The mean monthly exercise measurement rate was high (68.5–81.6%, Fig. 2a). SDSCA results were good, with significant improvement relative to the control group (6-month, *P* = 0.029; 18-month, *P* = 0.022; Fig. 2d, g), indicating improvements in lifestyle compliance. Daily step counts (monthly means of 7552–8693, Fig. 3) stayed consistent throughout the intervention period. Although measurement rates decreased over the course of the intervention, falling from 80.6% to 68.5%, the SDSCA results show that the intervention increased exercise levels, with the effect persisting after the intervention (at month 18).

Similarly, the intervention uses monitoring and feedback to increase blood glucose monitoring, with results that also show a significant improvement relative to the control group (6-month, *P* < 0.001; 12-month, *P* < 0.001). Measurement rates were high, with monthly means falling from 73.4% to 59.0% over the intervention. SDSCA results show improvement (Fig. 2b, e, h), though the improvement disappeared after the intervention (at month 18). Finally, for diet, monthly mean meal measurement rates were low and declined from 54.0% to 37.2% over the intervention, and the intervention did not show significant improvement relative to the control group (Fig. 2c, f, i).

The intervention group’s monthly mean study retention rate at 12 months was very high, at 93.9% (62/66), while monthly mean engagement rate with DialBetesPlus was high, at 81.6% at 12 months. Average daily measurement rate of body weight fell from 77.5% to 61.7%, with measurement of morning BP falling from 71.5% to 59.1%. There were no dropouts in the intervention group due to DialBetesPlus systems failures.

### Effect of intervention on health

**Fig. 4:**
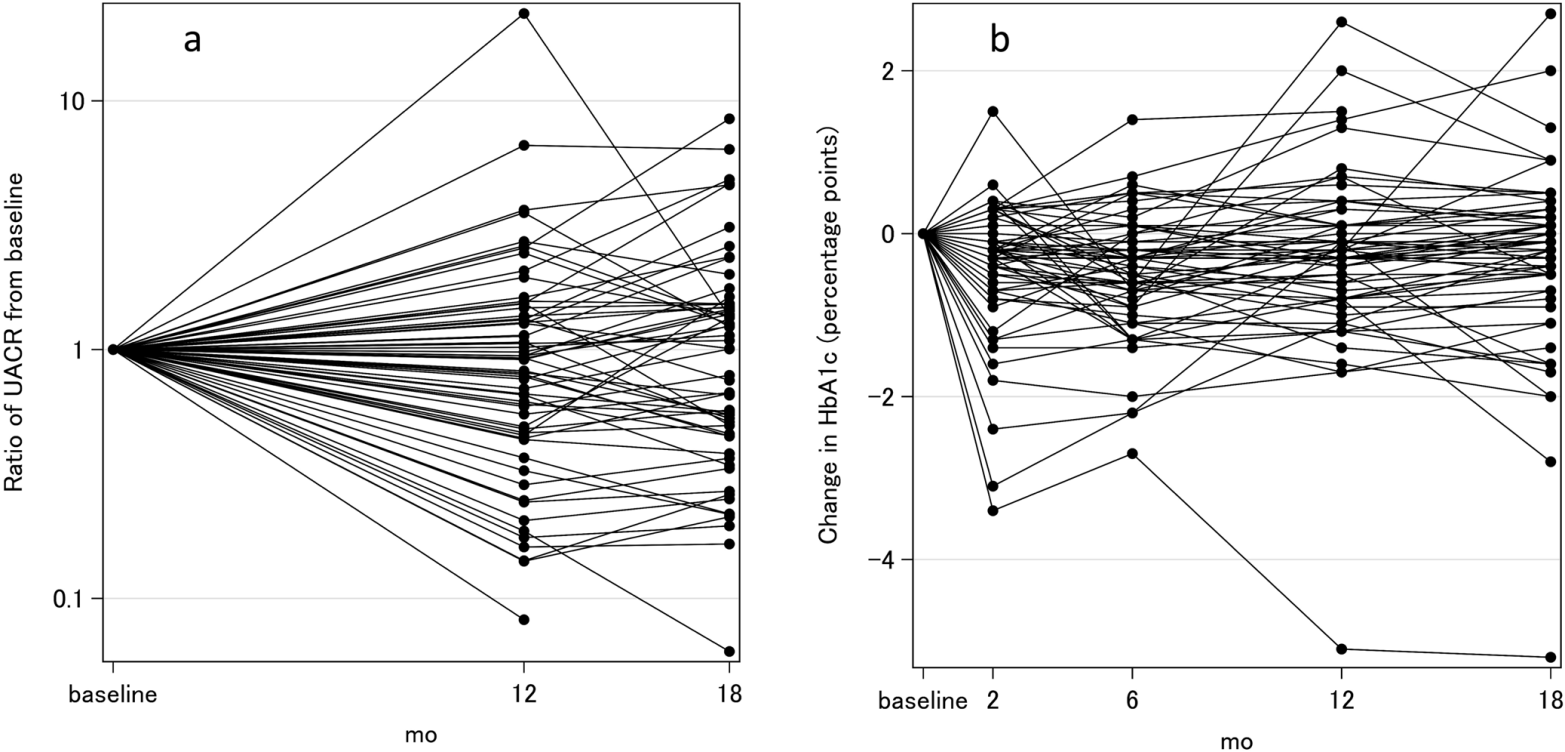
Change in intervention group UACR and HbA1c. Data is relative to baseline measurements for each patient. **a** The ratio of UACR to baseline UACR. **b** The change in HbA1c (in percentage points) relative to baseline HbA1c.

The intervention group saw a range of changes in UACR, with most patients improving (Fig. 4a). The primary outcome, change in UACR after 12 months, had a statistically and clinically significant between-group difference in change of −28.8% (*P* = 0.029) (Table 2). The intervention group had nearly twice as many patients with a reduction of ≥30% of baseline^24^ UACR (25/62 (40.3%) vs. 12/59 (20.3%), relative risk = 1.98, *P* = 0.019.) For secondary outcomes, HbA1c had a similar range of changes (Fig. 4b), with a statistically and clinically significant between-group difference in change of −0.32 points (*P* = 0.041) (Table 2). HDL-C had a statistically and clinically significant improvement compared to the control group (*P* = 0.041). While the intervention group had better eGFR (between-arm difference of −2.3 mL/min/1.73m^2^), suggesting better kidney function, the difference did not reach statistical significance (*P* = 0.141). Other parameters showed no significant difference between the two groups. Improvements in UACR and HbA1c continued at the month 18 follow-up, although improvements in the control group made the difference no longer significant.

**Table 2:** Summary of outcomes at 12 months of intervention

	Control^a^		Intervention^b^		Difference^c^		*P*^d^
UACR (%)	15.8^e^	(−3.3, 38.6)	−17.6^e^	(−35.8, 5.8)	−28.8 ^f^	(−47.5, −3.5)	0.029
HbA1c (%)	0.05	(−0.12, 0.22)	−0.28	(−0.54, −0.01)	−0.32	(−0.64, −0.01)	0.041
FPG (mg/dL)	−8.8	(−19.0, 1.4)	−4.9	(−18.3, 8.5)	3.9	(−12.8, 20.6)	0.643
BMI (kg/m^2^)	−0.3	(−0.6, 0.0)	−0.7	(−1.0, −0.4)	−0.4	(−0.8, 0.0)	0.074
Systolic BP (mmHg)	2.7	(−1.7, 7.1)	0.2	(−4.8, 5.2)	−2.4	(−9.0, 4.2)	0.465
Diastolic BP (mmHg)	−2.1	(−4.9, 0.8)	−2.1	(−5.4, 1.2)	0.0	(−4.3, 4.3)	0.995
eGFR (mL/min/1.73m^2^)	−0.9	(−2.7, 0.9)	−3.2	(−5.8, −0.7)	−2.3	(−5.5, 0.8)	0.141
LDL-C (mg/dL)	−2.9	(−8.8, 2.9)	−1.3	(−5.9, 3.3)	1.7	(−5.7, 9.0)	0.656
HDL-C (mg/dL)	−0.3	(−2.6, 2.0)	2.8	(0.8, 4.7)	3.1	(0.1, 6.0)	0.041
Triglycerides (mg/dl)	−6.2	(−40.6, 28.1)	−32.0	(−56.2, −7.7)	−25.7	(−67.4, 15.9)	0.223

Change from baseline and group difference are expressed as mean (95% CI). *P* values were assessed by t test.

^a^change between month 12 and baseline in the control group.

^b^change between month 12 and baseline in the intervention group.

^c^between-group difference of mean change.

^d^compared by t test.

^e^geometric mean of ratio of month 12 to baseline, expressed as percent change.

^f^ratio of change in intervention group over ratio of change in control group, expressed as percent change.

To exclude the possible influence of baseline differences between the control and intervention groups in coadministration of drugs known to improve albuminuria, we performed a *post hoc* assessment of outcomes via analysis of covariance (ANCOVA) using as covariates the baseline value of the outcome under analysis along with the use of each of GLP-1 receptor agonists, SGLT-2 inhibitors, ACE inhibitors, and ARBs at baseline, along with a subgroup analysis of UACR eliminating 14 patients (ten intervention, four control) whose use of these albuminuria-related drugs intensified during the first six months of the study. The results (Supplementary Table 1, Supplementary Figure 1) show improvements that are very similar to those in Table 2, with similar improvements in BMI now reaching statistical significance (*P* = 0.045). Removing outliers from the analysis as a sensitivity test also produced similar results.

Our *post hoc* exploratory multivariate linear regression analysis of change in log-UACR (Supplementary Table 2) showed statistical significance for the resulting model’s variables of baseline value of log-UACR (*P* < 0.001), the change in HbA1c (*P* = 0.003), and the change in sBP (*P* = 0.010). There was no statistical significance for the variable of change in blood glucose. When using the mean change in inputs, only the change in HbA1c achieved a clinically significant model impact, with a −10.8% change in UACR from a change in HbA1c of −0.28 points.

Since the multivariate regression analysis of log-UACR suggests that change in HbA1c was a key driver, we did a similar *post hoc* multivariate regression analysis of change in HbA1c (Supplementary Table 3). The resulting model shows statistical significance for all four variables: baseline value of HbA1c (*P* < 0.001), daily steps (*P* = 0.045), change in blood glucose (*P* = 0.002), and change in BMI (*P* = 0.011). Using the mean values for the inputs results in clinically significant (−0.35 point change from 7560 steps) and weaker (−0.15 point change from −0.7 kg/m^2^ change in BMI, 0.07 point change from 4.07 mm/dL change in glucose) changes in modeled HbA1c percentage.

These regression analyses, while not conclusive, suggest a primary biological mechanism for the results:

1. The intervention improved HbA1c via improvements in exercise.
2. The physiological changes reflected in this improvement in HbA1c in turn led to an improvement in UACR.

There were no all-cause mortality events, composite cardiovascular outcomes, composite kidney endpoints, severe hypoglycemic events, or adverse events. We found no significant between-arm changes in ADDQoL.

## Discussion

In this study, we employed albuminuria as a surrogate endpoint for predicting kidney and cardiovascular outcomes. Evidence suggests that albuminuria is closely associated with significant clinical endpoints and acts as an early indicator of disease progression, making it a valid and widely used surrogate^5,6,9^. However, surrogate endpoints have inherent limitations, including their inability to directly measure clinical outcomes like ESKD, creatinine doubling, and cardiovascular death. Consequently, future research is necessary to determine whether lifestyle interventions via mHealth can genuinely impact these hard endpoints beneficially.

Studies have shown association between physical activity and improvements in UACR and kidney function, including reduction to the risk of renal failure^25^. Exercise-based lifestyle interventions have shown a causal link to improved UACR^26–29^. To our knowledge, the current study is the first to show significant improvements in UACR from an mHealth-based lifestyle intervention.

The relative UACR change of −28.8% (−32.3% using ANCOVA) in the present study is comparable in magnitude to 17–32%^30,31^, 2–39%^32^, and 29%^33^ changes from reported drug-based albuminuria-targeted interventions, and this magnitude of change seems to be clinically relevant. However, the mechanism underlying the improvement in UACR seen in this intervention may differ from that of drug-based interventions, and we cannot definitively conclude that the intervention improved long-term kidney health. We are unaware of any studies linking a lifestyle intervention’s improvement in UACR to long-term kidney health, and this is an area that needs further work.

Improved HbA1c has a proven impact on kidney health, and the study’s relative HbA1c change of −0.32 points corresponds to approximately one additional year of sight, absence of kidney failure, absence of lower extremity amputation, and life^34^. The findings suggest that the mHealth intervention employing DialBetesPlus was effective in delaying the progress of DKD.

Our somewhat elderly participants could use the system without burden. Our high engagements are promising, as initial engagement with mHealth applications is closely related to long-term engagement^35^. Automated pedometer counting had the highest engagement, though measurement rates declined with time. A previous multicomponent mHealth trial showed similar decreases with time^36^. Personalization of mHealth content, social and gamification features, and personal support have been shown to improve adherence to mHealth^37^. There is a need to further explore mechanisms to improve long-term engagement.

The mHealth intervention by DialBetesPlus seems to have led to increased exercise, as measured via the SDSCA exercise score, and may have improved insulin sensitivity, reducing cardiovascular risk and improving albuminuria^38–40^. We do not have a baseline step count, but the high step count, 7000–8000 (7552 ± 4449) steps per day, corresponds well with the recommended 150 min per week of activity to prevent all-cause mortality^41–44^ and results showing that increasing to 6000–8000 daily steps decreased mortality among adults aged 60 years and older. Blood glucose monitoring had good engagement but did not definitively lead to improved outcomes in this study. The diet feature had low engagement and SDSCA scores indistinguishable from those of the control group, and we conclude that this feature was not effective as currently implemented. In terms of the TPB framework, the intervention did not succeed in improving dietary control, presumably because, relative to the comparatively simple choice of whether or not to exercise, diet involves complicated choices throughout the day balancing practicalities (for instance, the availability of prepared food), desirabilities (taste, satisfying hunger, etc.) and multiple dimensions of nutrition (calories, macronutrients, fiber, salt, etc.). There seems to be a need for more specific and individualized information to support patient control over dietary behavior. Future work to improve the engagement with and effectiveness of features addressing meal monitoring and assess any resulting health improvements is warranted.

We did not find significant improvement in BP, despite increased exercise, perhaps because of limited room for improvement given fairly well-controlled baseline BP.

We are unable to definitively evaluate which features contributed most to improved outcomes, although we expect improvements are related to the features with the highest engagement. Features could have interacted with one another. The addition of a self-management support system could have enhanced patient engagement, a critical component of successful treatment of T2D and DKD^45,46^, and may have improved patient focus on multifactorial intervention^47^.

The intervention is general in nature and may be readily extended to other patient populations. The Japanese guidelines for lifestyle modifications are similar to those in other countries, and we expect this intervention would show similar positive results, including improved albuminuria, for diabetes patients in other countries. More broadly, the intervention could apply for any patient population who would benefit from these lifestyle modifications, not just diabetes patients. At present, the DialBetesPlus application is a research product, not production software, and applying this intervention more broadly, whether for diabetes patients in Japan or for a wider population, would likely require an industry partner who would develop a full production application.

Use and changes in use of RAAS inhibitors, GLP-1 receptor agonists, and SGLT-2 inhibitors were allowed during the study, potentially confounding any effect of the intervention on UACR. Our analysis adjusting for usage prior to the intervention and for intensification during the intervention showed similar UACR results, reducing the chance that these agents partially account for our results. Similarly, our exploratory multivariate regression analysis did not show that intensification of diabetes drugs was a significant driver of change in HbA1c. Our lack of restriction on concurrent diabetes management allowed the study to be conducted in a condition close to that of the real world.

The study has limitations. The study was conducted among a Japanese population, and there are differences between Japanese and other populations in lifestyles and in the pathophysiology of T2D and DKD. The study was limited to patients able to use mobile phones, so there may be biases due to users’ digital literacy. Participants were not blinded to randomization, so social desirability bias may have affected the results. The study’s focus on patients with mild DKD may limit generalizability to those with more advanced DKD. Albuminuria is only one aspect of DKD—further research on the intervention’s impact on broader aspects of kidney function is warranted. Our use of single UACR measures at each time point, first-morning void as a good alternative to measuring 24-h urinary albumin excretion^48^, may cause a bias toward the null. Many factors influence UACR, not all of which were captured in these analyses. For example, we were unable to capture the effects of changes in albuminuria-related drugs between six and 12 months, and our measurements of diet were ineffective, a clear weakness of our study. As clinical studies of DKD with digital health are relatively new, we were unable to use previous studies to fully plan statistical analyses or develop hypotheses, and therefore some of the analyses were *post hoc* and exploratory. These results should be considered hypotheses.

This study is, to our knowledge, the first to show that the addition of a real-time self-management mHealth system to standard care reduced albuminuria. Glycemic control improved and was comparable to that of other mHealth interventions incorporating coaching from health care providers^49,50^. This intervention does not require healthcare personnel, so it has great potential for scalability. As the use of smart phones and the mobile internet becomes ubiquitous in daily life, a self-management support mHealth system is recommended to reduce the lifestyle risk factors of DKD patients. Future studies are needed to improve long-term engagement with the mHealth system and to develop effective implementations within the broader healthcare system.

## Methods

### Participant inclusion criteria and recruitment

Inclusion criteria were outpatients to the eight registered medical institutions who were diagnosed with type 2 diabetes with moderately increased albuminuria (UACR: 30–299 mg/g creatinine). The institutions (Supplementary Table 4) as well as inclusion and exclusion criteria (Supplementary Table 5) were described in detail previously^51^. Medication therapy (antidiabetic, lipid lowering, and antihypertensive) could be adjusted as needed during the study consistent with standard care.

After asking all enrolled patients to use DialBetesPlus for two weeks, we retained as eligible for the study those who used the application and devices for at least seven days during the two weeks. (We judged that the two-week trial would have no significant effect on outcomes.) We then used SAS 9.4 to randomize eligible participants one-to-one to either the intervention or the control group using the covariance-adaptive randomization by minimization method with random element of 0.75 to ensure covariance balance for age (≤40, 41–59, ≥60), sex, and UACR (<100, ≥100), stratified by institution^52^. Our protocol defined participants to be dropouts if there was no data input after three weeks or if the research team lost contact with the participants.

### DialBetesPlus design

The DialBetesPlus intervention seeks to change patient behavior by improving adherence to existing prescribed lifestyle modifications, rather than introducing a new treatment. The desired behavior changes are increased exercise and a move to diabetes-appropriate dietary choices, and these behavior changes can be viewed using the framework of the Theory of Planned Behavior (TPB). TPB is a behavior change theory that has seen wide use and success in physical behavior interventions^53^. At a high level, the TPB framework models behavior as driven by the individual’s intention to perform the behavior and by their control (both perceived and actual) over the behavior. Using the TPB framework, the DialBetesPlus intervention does not target intention, based on an assumption that most patients are well motivated to address this serious disease. Rather, the intervention seeks to strengthen both perceived and actual control by giving patients timely and detailed information on behavior (exercise and diet) as well as intermediate health outcomes (blood glucose, BP, and body weight). This feedback allows patients to see which individual behavioral decisions lead to success in achieving the desired behavior and intermediate health outcomes, with the goal of ultimately improving glycemic control and slowing the progression of kidney disease.

**Fig. 5:**
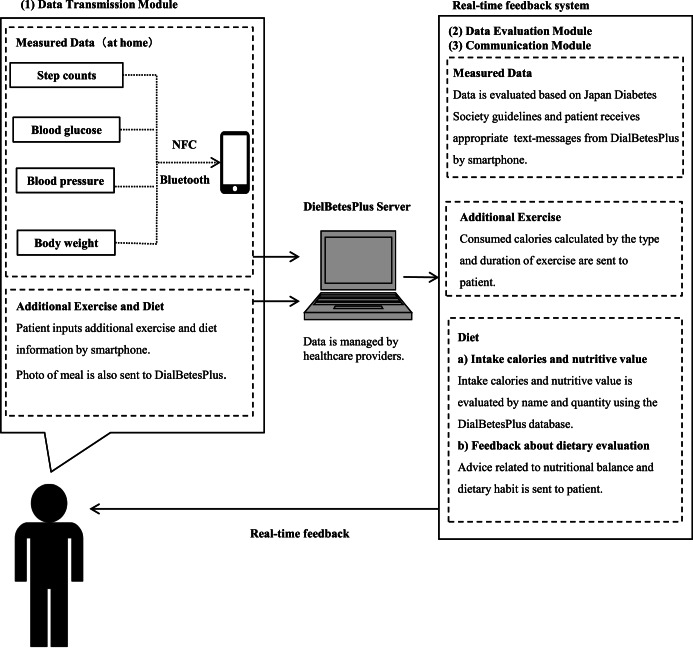
Summary of DialBetesPlus. The DialBetesPlus application tracks steps, blood glucose, blood pressure, body weight, additional exercise, and diet. It sends the data to the DialBetesPlus server, where the data is evaluated against guidelines and tailored feedback is provided to the patient through the application. NFC: near field communications. (This figure was published in JMIR Research Protocols (and can be reproduced) under the terms of Creative Commons Attribution 4.0 license^51^).

**Fig. 6:**
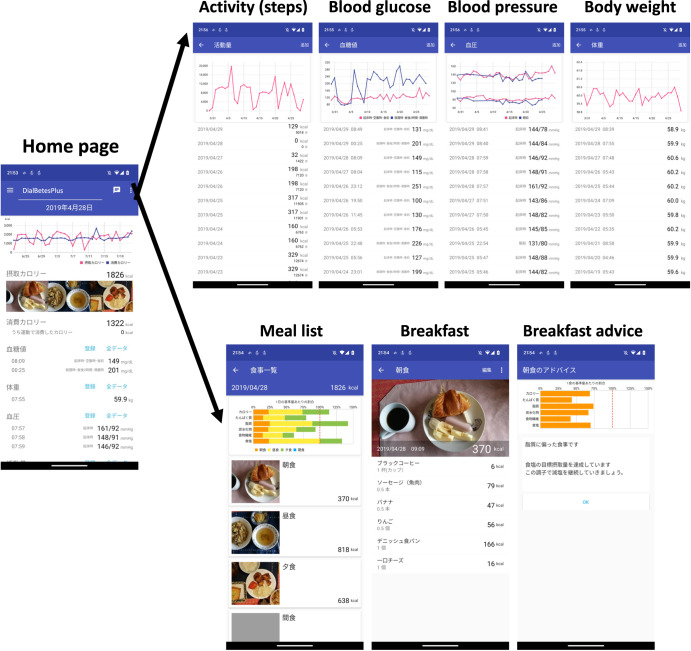
Example DialBetesPlus Feedback. Users receive feedback on steps, blood glucose, blood pressure, body weight, additional exercise, and diet.

Using DialBetesPlus (Fig. 5)^51^, patients measure daily step counts, blood glucose, BP, and body weight at home. Data is transferred from each device to the DialBetesPlus smartphone application and immediately sent to the DialBetesPlus server and evaluated following the target values of the Japan Diabetes Society (JDS) guidelines (http://www.fa.kyorin.co.jp/jds/uploads/Treatment_Guide_for_Diabetes_2016-2017.pdf, accessed on April 18, 2023). The step target is 8000 or more steps per day, and target maximum values are blood glucose levels of 110 mg/dl before breakfast and 140 mg/dl at bedtime, and BP of 125/75 mmHg. Feedback is sent to the participant’s smartphone (Fig. 6). In addition, patients enter the content and quantity of their meals by text message with a photograph of the meal. Software on the server calculates nutrient intake and consumed calories, generates JDS-based advice for improving dietary habits, and sends the measurements and advice to the smartphone. Patients are also able to enter the type and duration of exercise that was not recorded on the pedometer. Patients can review their data in the forms of graphs.

### Study design

The study was a prospective, randomized, open-label, multicenter clinical trial. M.M. generated the random allocating sequence used to randomize the participants into either the intervention or control group in a one-to-one fashion based on albuminuria levels, gender, and age. After randomization, the principal investigator (K.W.) communicated the assignment to the study collaborators (Y.K., K.M., Y.T., S.K., A.I., S.S., T.M., and T.T.) who enrolled the participants. Patients assigned to the intervention group used DialBetesPlus for 12 months, with a post-intervention follow-up at month 18. We provided the intervention group patients with an NFC-enabled blood glucose meter (MS-FR201B; Terumo), a Bluetooth-enabled BP monitor (HEM-7271T; Omron), an NFC-enabled pedometer (MT-KT02DZ; Terumo), and a Bluetooth-enabled scale (HBF-255T; Omron). The devices were paired with a smartphone (Arrows F-02H: Fujitsu or Galaxy Note3 SC-01F: Samsung) prepared with the DialBetesPlus application. We provided participants in the control group with a home BP monitor as part of standard care. We established each patient’s baseline by collecting background demographic information, physical parameters (BMI and BP), blood test data, UACR by first morning void, medication regimen, and patients’ QOL and self-care evaluation via questionnaire, with follow-up studies at two, six, 12 and 18 months. (UACR was obtained only at baseline, month 12, and month 18.).

### Outcome measures

The primary outcome was change after 12 months in UACR. Secondary outcomes (Supplementary Table 6) included change after 12 months in blood test parameters, physical parameters (BMI and BP), lifestyle habits and diabetes self-care assessment, and QOL, along with changes in medication therapy, study retention rate, engagement rate, and daily measurement rate of DialBetesPlus. We tracked any DialBetesPlus system failures. To ensure safety, we monitored the number of hypoglycemic events and other adverse events.

### Research ethics

We conducted this study in compliance with the Helsinki Declaration. The Research Ethics Committee of the Graduate School of Medicine, the University of Tokyo and related facilities approved the study protocol and informed consent form. We registered the study in the University Hospital Medical Information Network Clinical Trial Register (UMIN000033261). We obtained written informed consent from all participants prior to the study.

### Statistical analysis

We calculated the required sample size of 64, based on our target of 80% power at a two-sided significance level of 0.05 and assuming a between-arm difference of change in UACR of 100 mg/gCre with a standard deviation (SD) of 200^54,55^. Assuming a 20% loss rate, we required 80 initial subjects per group.

The study uses the Full Analysis Set of all patients after randomization for which data were obtained at least once. Data on patients’ characteristics are presented as mean and SD for continuous variables that follow a normal distribution, and median and interquartile ranges for continuous variables with a non-normal distribution. Categorical variables are presented as frequency and proportion. We compared continuous variables using a two-sided t-test with a significance level of 5% or a Wilcoxon rank sum test. For UACR, we used logarithmically transformed data (log-UACR) due to a skewed distribution. As the levels of missing data were low, with 4.0% (5/126) of patients missing UACR data and 4.0% missing HbA1c data, we used standard methods^56,57^ without imputation for missing data. A secondary analysis using imputation (multiple imputation by chained equations procedure and 100 imputations for missing data) showed similar results (results not shown.) We analyzed categorical variables using Fisher’s exact test or the Chi square test, changes in J-SDSCA and JP-ADDQoL scores using t-test, and changes in medication regimen at 12 months using Fisher’s exact test.

We performed *post hoc* sensitivity analyses to assess the effect of clinically relevant covariates on outcomes and explore possible mechanisms underlying our results. To exclude the possible influence of baseline differences between the groups in coadministration of drugs known to improve albuminuria, we assessed outcomes via ANCOVA using as covariates the baseline value of the outcome under analysis along with the use at baseline of each of GLP-1 receptor agonists, SGLT-2 inhibitors, Angiotensin-converting enzyme (ACE) inhibitors, and Angiotensin receptor blockers (ARBs). We also performed a subgroup analysis of the ANCOVA results for UACR wherein we eliminated patients whose use of these albuminuria-related drugs intensified during the first six months of the study.

To address our secondary objective of identifying physical/biological parameters most strongly related to UACR and HbA1c reduction, we conducted exploratory multivariate linear regressions. Based on previous research and clinical judgment, we included candidate variables with established or presumed importance in UACR (13 variables) and HbA1c (13 variables) (Supplementary Table 7). We used the best subset method, fitting separate regression models for all combinations of up to four variables, based on 66 patients and the criterion of at least 15 patients per variable^58^, to determine which model is best^59,60^ based on the criteria of highest adjusted R^2^.

We defined statistical significance as a two-sided *P*-value less than 0.05 and used SAS (version 9.4 M7; SAS Institute Inc) for statistical analysis.

### Reporting summary

Further information on research design is available in the Nature Research Reporting Summary linked to this article.

### Supplementary information

Supplementary material

Reporting Summary

## Data Availability

The data that support the findings of this study are available from the corresponding author upon reasonable request for non-commercial purposes.

## Code availability

The code used for the analysis in this study is based on a common procedure used in the statistical analysis software SAS. No mathematical algorithms or/and original code are used.
