# Author Correction: Effectiveness of DialBetesPlus, a self-management support system for diabetic kidney disease: Randomized controlled trial

**DOI:** 10.1038/s41746-024-01158-w

**Published:** 2024-06-15

**Authors:** Kayo Waki, Mitsuhiko Nara, Syunpei Enomoto, Makiko Mieno, Eiichiro Kanda, Akiko Sankoda, Yuki Kawai, Kana Miyake, Hiromichi Wakui, Yuya Tsurutani, Nobuhito Hirawa, Tadashi Yamakawa, Shiro Komiya, Akihiro Isogawa, Shinobu Satoh, Taichi Minami, Tamio Iwamoto, Tatsuro Takano, Yasuo Terauchi, Kouichi Tamura, Toshimasa Yamauchi, Masaomi Nangaku, Naoki Kashihara, Kazuhiko Ohe

**Affiliations:** 1 Department of Biomedical Informatics, Graduate School of Medicine, The University of Tokyo, Tokyo, Japan; 2 Department of Planning, Information and Management, University of Tokyo Hospital, Tokyo, Japan; 3 Department of Diabetes and Metabolic Diseases, Graduate School of Medicine, The University of Tokyo, Tokyo, Japan; 4 Department of Metabolism and Endocrinology, Akita University Graduate School of Medicine, Akita, Japan; 5 Department of Medical Informatics, Center for Information, Jichi Medical University, Shimotsuke, Japan; 6 Medical Science, Kawasaki Medical School, Kurashiki, Japan; 7 Department of Medical Science and Cardiorenal Medicine, Yokohama City University Graduate School of Medicine, Yokohama, Japan; 8 Endocrinology and Diabetes Center, Yokohama Rosai Hospital, Yokohama, Japan; 9 Department of Nephrology and Hypertension, Yokohama City University Medical Center, Yokohama, Japan; 10 Department of Endocrinology and Diabetes, Yokohama City University Medical Center, Yokohama, Japan; 11 Division of Diabetes, Mitsui Memorial Hospital, Tokyo, Japan; 12 Department of Endocrinology and Metabolism, Chigasaki Municipal Hospital, Chigasaki, Japan; 13 Department of Diabetes and Endocrinology, Saiseikai Yokohamashi Nanbu Hospital, Yokohama, Japan; 14 Department of Nephrology and Hypertension, Saiseikai Yokohamashi Nanbu Hospital, Yokohama, Japan; 15 Department of Diabetes and Endocrinology, Fujisawa City Hospital, Fujisawa, Japan; 16 Department of Endocrinology and Metabolism, Yokohama City University Graduate School of Medicine, Yokohama, Japan; 17 Division of Nephrology and Endocrinology, Graduate School of Medicine, The University of Tokyo, Tokyo, Japan; 18 Department of Nephrology and Hypertension, Kawasaki Medical School, Kurashiki, Japan

**Keywords:** Type 2 diabetes, Lifestyle modification, Kidney diseases

Correction to: *npj Digital Medicine* 10.1038/s41746-024-01114-8, published online 27 April 2024

In this article Kayo Waki and Mitsuhiko Nara should have been denoted as equally contributing authors. The original article has been corrected.

