# Supplementary material for: Effectiveness of DialBetesPlus, a self-management support system for diabetic kidney disease: Randomized controlled trial
Source: NPJ Digit Med. 2024 Apr 27;7:104. doi: 10.1038/s41746-024-01114-8 (PMC11055918; doi:10.1038/s41746-024-01114-8)
Supplement: Supplementary file 1 — Supplementary material [file 41746_2024_1114_MOESM1_ESM.pdf]

SUPPLEMENTARY MATERIAL

Supplementary Table 1 | Summary of ANCOVA outcomes at 12 months of intervention

	Control[a]		Intervention[b]		Difference[c]		*P*[d]
UACR (%)	41.6[e]	(2.8, 95.1)	-4.2[e]	(-26.5, 24.9)	-32.3[f]	(-49.2, -9.8)	0.008
UACR (%) for subgroup[g]	48.4[e]	(8.8, 102.5)	5.1[e]	(-19.7, 37.5)	-29.2[f]	(-46.1, -7.0)	0.013
HbA1c (%)	0.08	(-0.24, 0.40)	-0.24	(-0.50, 0.03)	-0.32	(-0.60, -0.03)	0.029
FPG (mg/dL)	-4.6	(-19.7, 10.6)	1.3	(-11.3, 13.8)	5.8	(-7.9, 19.6)	0.407
BMI (kg/m2)	-0.1	(-0.6, 0.4)	-0.5	(-0.9, -0.1)	-0.4	(-0.8, 0.0)	0.045
Systolic BP (mmHg)	7.2	(0.8, 13.6)	3.8	(-1.5, 9.2)	-3.4	(-9.2, 2.4)	0.256
Diastolic BP (mmHg)	0.9	(-3.3, 5.2)	-0.7	(-4.2, 2.9)	-1.6	(-5.5, 2.3)	0.416
eGFR (mL/min/1.73m2)	-0.6	(-4.0, 2.8)	-2.3	(-5.1, 0.5)	-1.7	(-4.7, 1.3)	0.271
LDL-C (mg/dL)	4.6	(-3.0, 12.3)	2.9	(-3.4, 9.2)	-1.7	(-8.5, 5.0)	0.616
HDL-C (mg/dL)	-1.3	(-4.5, 2.0)	2.0	(-0.6, 4.7)	3.3	(0.4, 6.2)	0.025
Triglycerides (mg/dl)	5.0	(-31.8, 41.7)	-16.3	(-46.9, 14.3)	-21.3	(-54.3, 11.8)	0.207

Change from baseline and group difference are expressed as mean (95% CI) estimated by ANCOVA using the baseline of the variable being analyzed and medication usage (whether each of GLP-1 receptor agonists, SGLT-2 inhibitors, ACE inhibitors, and ARBs are used or not). ANCOVA; analysis of covariance.

[a]change between month 12 and baseline in the control group

[b]change between month 12 and baseline in the intervention group

[c]between-group difference of mean change

[d]compared by t test

[e]geometric mean of ratio of month 12 to baseline, expressed as percent change

[f]ratio of change in intervention group over ratio of change in control group, expressed as percent change

[g]Subgroup was defined as the group excluding 14 patients (10 intervention, 4 control) whose medications for GLP-1 receptor agonists, SGLT-2 inhibitors, ACE inhibitors or ARBs were intensified during the first six months of the intervention period

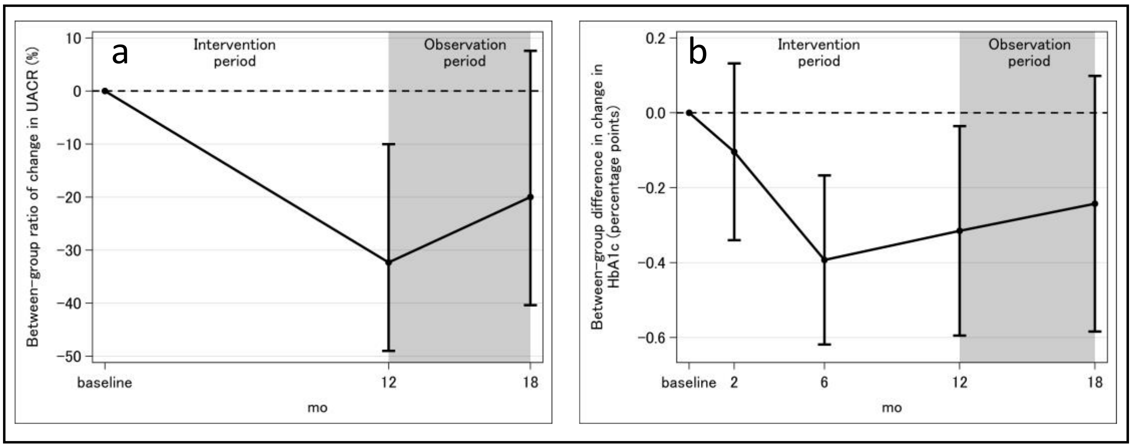

Supplementary Figure 1 | UACR and HbA1c ANCOVA results. The analysis uses as covariates the baseline value of the variable under analysis along with the use of each of GLP1, SGLT2, ACE, and ARB at baseline. a) The between-arm ratio of the ratio of UACR to baseline shows 32.3% improvement at month 12. b) The between-arm difference in change of HbA1c from baseline shows

0.32 percentage points of improvement at month 12.

Error bars represent 95% confidence interval.

Supplementary Table 2 | Linear regression for change in log UACR in the intervention group – result of the best subset method with up to four variables

R^2=0.466

Variable	Estimate	95% CI		*P*
Intercept	1.148	0.470	1.826	0.001
LogUACR[a] at baseline	-0.381	-0.570	-0.192	<0.001
Change in HbA1c (pts)	0.408	0.151	0.665	0.003
Change in blood glucose (mg/dL)[b]	-0.006	-0.017	0.006	0.325
Change in sBP (mmHg)[c]	0.033	0.008	0.057	0.010

[a]log of the ratio of UACR to a reference 1 mg/gCr

[b]change in self-monitored blood glucose as measured before breakfast between the first and the last three months of the intervention period

[c]change in self-monitored sBP as measured before breakfast between the first and the last three months of the intervention period

Supplementary Table 3 | Linear regression for change in HbA1c in the intervention group–result of the best subset method with up to four variables

R^2=0.369

Variable	Estimate	95% CI		*P*
Intercept	2.778	1.391	4.164	<0.001
HbA1c at baseline (%)	-0.340	-0.508	-0.171	<0.001
Steps (1000's)[a]	-0.045	-0.090	-0.001	0.045
Change in blood glucose (mg/dL)[b]	0.017	0.007	0.027	0.002
Change in BMI (kg/m^2)[c]	0.220	0.054	0.386	0.011

[a]average number of daily steps (in thousands of steps) of the last three months of intervention

[b]change in self-monitored blood glucose as measured before breakfast between the first and the last three months of the intervention period

[c]change in BMI between baseline and 12 months

Supplementary Table 4 | List of participating investigators

List of participating hospitals and physicians

The University of Tokyo Hospital			
Naoko Arakawa	Yuta Hiraike	Yusuke Hirota	Hirofumi Honma
Jun Hosoe	Masahiko Iwamoto	Tomoya Kawaguchi	Yuka Kobayashi
Masaomi Miura	Sachiko Okazaki	Yoshitaka Sakurai	Takayoshi Sasako
Tomonobu Sawada	Minaka Takakura	Mikio Takanashi	Satoru Takase
Masaki Tanaka	Gotaro Toda	Hironori Waki	Toshimasa Yamauchi
Kana Miyake	Kayo Waki		

Yokohama City University Hospital			
Ryoichi Akamatsu	Masanori Arai	Tatsuo Hashimoto	Rina Hiratsuka
Takahiro Iijima	Ryota Inoue	Yuzuru Ito	Masayo Kimura
Ryu Kobayashi	Rieko Kunishita	Mayu Kyohara	Hirotatsu Nakaguchi
Naohito Okami	Tomoko Okuyama	Sumire Sunohara	Sakiko Terui
Yu Togashi	Kazushi Uneda	Shingo Urate	Machiko Yabana
Yoshihiko Yamada	Yuki Kawaki	Hiromichi Wakui	Shiro Komiya

Yasuo Terauchi	Kouichi Tamura		
**Yokohama Rosai Hospital**			
Rei Hirose	Takuhei Hitsuwari	Hirofumi Horikoshi	Yoshitomo Hoshino
Masahiro Ichikawa	Sho Katsuragawa	Haremaru Kubo	Takashi Sunouchi
Tomoko Takiguchi	Takahiro Yamane	Yuya Tsurutani	
**Yokohama City University Medical Center**			
Tatsuya Haze	Masahiro Ichikawa	Yoshinobu Kondo	Akeo Ohira
Yasuyuki Sugiura	Nubuhito Hirawa	Tadashi Yamakawa	
**Chigasaki Municipal Hospital**			
Masanori Hasebe	Taichi Suezono	Hitoshi Tamaki	Shinobu Satoh
**Saiseikai Yokohamashi Nanbu Hospital**			
Akiko Kameda	Tamio Iwamoto	Taichi Minami	
**Mitsui Memorial Hospital**			
Akihiro Isogawa			
**Fujisawa City Hospital**			
Tatsuro Takano			

List of pharmacists and dieticians from Nihon Chouzai Co, Ltd.

Pharmacists

Keiichi Chin
Yoshihiko Kimura
Seiji Sakumoto,
Dieticians
Hinano Asahigata
Yuma Tanaka

Supplementary Table 5 | Inclusion and Exclusion Criteria

**Inclusion Criteria**
• Diagnosed with T2DM<br>• HbA1C 6.5% or more<br>• Between 20 and 75 years of age<br>• BP lower than 180/110 mmHg<br>• eGFR 45mL/min/1.73m^2 or more<br>• Two detected instances of moderately increased albuminuria (30-299 mg/g creatinine) in spot urine samples prior to study enrollment

- BMI of 22 kg/m^2 or more
- No history of severe hypoglycemia requiring additional medical support
- No history of the following symptoms indicating hypoglycemia within the last 3 months: palpitations, tremors, dizziness, anxiety, loss of consciousness, sweating, facial pallor, tachycardia, headache, sleepiness, blurred vision, or convulsions
- Regular patients of hospitals listed in Supplementary Table 1
- Signatories of the informed consent form

Exclusion criteria

- Use of cardiac pacemaker
- Hyperthyroidism diagnosis, under medication other than thyroid hormone supplementation in the last year
- Medical instability or exercise restriction as ordered by a physician, with autoimmune, heart, liver, digestive, neurological, or respiratory disease
- Hb less than 10 g/ dL
- Albumin 3.0 g/dL or less

- eGFR less than 45mL/min/1.73m^2
- Those with preproliferative diabetic retinopathy within one year of signing consent forms
- Inability to exercise
- Pregnancy, potential planned pregnancy, or lactating
- Participation in other clinical trials
- Under a diet that restricts protein
- Judged as ineligible by doctor's discretion for other reasons

Supplementary Table 6 | Secondary Outcomes

**Outcome**	**Details**
Blood glucose control indicators	HbA1c, fasting plasma glucose (FPG)
Kidney function indicators	Creatinine, estimated glomerular filtration rate (eGFR)
Lipid metabolism indicators	High-density lipoprotein cholesterol (HDL-C), low-density lipoprotein cholesterol (LDL-C), triglycerides (TG)

Change in medication therapy (antidiabetic, lipid lowering and antihypertensive)	Treatment intensification, no change, or treatment deintensification, as assessed by five diabetes specialists
Changes in lifestyle habits and self-management	Via the Japanese version of the Summary of Diabetes Self-Care Activities Measure (J-SDSCA) [1,2]
Quality of life (QOL)	Via the Japanese version of the Audit of Diabetes-Dependent Quality of Life (JP-ADDQoL) [3,4]
Overall outcomes	All-cause mortality, composite cardiovascular outcome, composite kidney endpoints, safety
Study retention rate	Percentage of subjects who remained in the study from the time the intervention was allocated until the end of intervention
Engagement rate	Percentage of subjects who logged at least one record of either BP, blood glucose, body weight or step count in DialBetesPlus in one day
Daily measurement rates	Percentage of measurements logged daily in

	DialbetesPlus for each of step count, blood glucose, BP, and body weight

Supplementary Table 7 | Candidate independent variables for exploratory multivariate linear regressions

Baseline log UACR (used in the UACR model only
Baseline HbA1c (used in the HbA1c model only)
Age
Sex
Smoking history (years)
Albuminuria-related drugs (SGLT-2 inhibitors, GLP-1 receptor agonists, ARBs, ACE inhibitors) intensified or not (used in the UACR model only)
Diabetes drugs intensified or not (used in the HbA1c model only)
Change in HbA1c (used in the UACR model only)
Change in average home blood glucose from the first three months to the last three months of the intervention
Change in BMI
Change in average home systolic blood pressure (sBP) from the first three months to the last three

months of the intervention
Change in LDL-C
Change in HDL-C
Change in TG
Average daily steps in the last 3 months of the intervention
Average blood glucose measurement rate in the last 3 months of the intervention (used in the HbA1c model only)

SUPPLEMENTARY REFERENCES

1. Toobert DJ, Hampson SE, Glasgow RE. The summary of diabetes self-care activities measure: results from 7 studies and a revised scale. Diabetes Care. 2000;23(7):943-50.

2. Daitoku M, Honda I, Okumiya A, et al. Validity and Reliability of the Japanese Translated “The Summary of Diabetes Self—care Activities Measure” J Japan Diab Soc 2006;49 (1):1-9.

3. Bradley C, Todd C, Gorton T, et al. The development of an individualized questionnaire measure of perceived impact of diabetes on quality of life: the ADDQoL. Qual Life Res. 1999;8(1-2):79-91.

4. Hirose AS, Fujihara K, Miyamasu F, et al. Development and evaluation of the Japanese version of the Audit of Diabetes-Dependent Quality of Life for patients with diabetes. Diabetol Int. 2016;7(4):384-390.
