# Supplementary material for: Effectiveness of DialBetesPlus, a self-management support system for diabetic kidney disease: Randomized controlled trial
Source: NPJ Digit Med. 2024 Apr 27;7:104. doi: 10.1038/s41746-024-01114-8 (PMC11055918; doi:10.1038/s41746-024-01114-8)
Supplement: Supplementary file 2 — Reporting Summary [file 41746_2024_1114_MOESM2_ESM.pdf]

nature portfolio

Corresponding author(s): Kayo Waki

Last updated by author(s): Dec 18, 2023

Reporting Summary

Nature Portfolio wishes to improve the reproducibility of the work that we publish. This form provides structure for consistency and transparency in reporting. For further information on Nature Portfolio policies, see our Editorial Policies and the Editorial Policy Checklist.

Statistics

For all statistical analyses, confirm that the following items are present in the figure legend, table legend, main text, or Methods section.

n/a	Confirmed	
☐	☒	The exact sample size (*n*) for each experimental group/condition, given as a discrete number and unit of measurement
☐	☒	A statement on whether measurements were taken from distinct samples or whether the same sample was measured repeatedly
☐	☒	The statistical test(s) used AND whether they are one- or two-sided<br>*Only common tests should be described solely by name; describe more complex techniques in the Methods section.*
☐	☒	A description of all covariates tested
☐	☒	A description of any assumptions or corrections, such as tests of normality and adjustment for multiple comparisons
☐	☒	A full description of the statistical parameters including central tendency (e.g. means) or other basic estimates (e.g. regression coefficient) AND variation (e.g. standard deviation) or associated estimates of uncertainty (e.g. confidence intervals)
☐	☒	For null hypothesis testing, the test statistic (e.g. *F*, *t*, *r*) with confidence intervals, effect sizes, degrees of freedom and *P* value noted<br>*Give P values as exact values whenever suitable.*
☒	☐	For Bayesian analysis, information on the choice of priors and Markov chain Monte Carlo settings
☒	☐	For hierarchical and complex designs, identification of the appropriate level for tests and full reporting of outcomes
☒	☐	Estimates of effect sizes (e.g. Cohen's *d*, Pearson's *r*), indicating how they were calculated

Our web collection on statistics for biologists contains articles on many of the points above.

Software and code

Policy information about availability of computer code

Data collection	No specific software or code was utilized for data collection.
Data analysis	SAS version 9.4 M7

For manuscripts utilizing custom algorithms or software that are central to the research but not yet described in published literature, software must be made available to editors and reviewers. We strongly encourage code deposition in a community repository (e.g. GitHub). See the Nature Portfolio guidelines for submitting code & software for further information.

Data

Policy information about availability of data

All manuscripts must include a data availability statement. This statement should provide the following information, where applicable:

- Accession codes, unique identifiers, or web links for publicly available datasets
- A description of any restrictions on data availability
- For clinical datasets or third party data, please ensure that the statement adheres to our policy

The data that support the findings of this study are available from the corresponding author upon reasonable request for non-commercial purposes.

Research involving human participants, their data, or biological material

Policy information about studies with human participants or human data. See also policy information about sex, gender (identity/presentation), and sexual orientation and race, ethnicity and racism.

Reporting on sex and gender	We use self-reported sex (biological attribute). No analysis by sex have been performed due to the small sample size of female.
Reporting on race, ethnicity, or other socially relevant groupings	This study did not use data on racial or ethnic classifications.
Population characteristics	The eligible 132 patients were randomly assigned between groups, yielding 66 (intervention) and 60 (control). The mean (SD) of age for the intervention and control groups was 58.6 (10.1) and 60.3 (8.7); the number (%) of male cases was 47 (71.2) and 46 (76.7); the geometric mean (SD) of UACR was 36.7 (2.9) and 30.1 (2.8) respectively.
Recruitment	Patients were recruited from patients who presented at each facility participating in the study. We believe that this is a randomized controlled trial and that there is no selection bias associated with recruitment.
Ethics oversight	The Research Ethics Committee of the Graduate School of Medicine, the University of Tokyo and related facilities approved the study protocol and informed consent form.

Note that full information on the approval of the study protocol must also be provided in the manuscript.

Field-specific reporting

Please select the one below that is the best fit for your research. If you are not sure, read the appropriate sections before making your selection.

☒ Life sciences ☐ Behavioural & social sciences ☐ Ecological, evolutionary & environmental sciences

For a reference copy of the document with all sections, see nature.com/documents/nr-reporting-summary-flat.pdf

Life sciences study design

All studies must disclose on these points even when the disclosure is negative.

Sample size	Sample size was caluculated 64 per group with 80% power, 5% two-sided singnificance level and assuming a between-arm difference of change in UACR of 100 mg/gCre with a standard deviation (SD) of 200. Assuming a 20% loss rate, we determined 80 patients per group.
Data exclusions	Using all patients with obtained least one data after randomization.
Replication	We can not replicate because this is a clinical study.
Randomization	We randomized eligible patient one to one using covariance-adaptive randomization by minimization method with random element of 0.75 to ensure covariance balance for age (≤40, 41-59, ≥60), sex, and UACR (<100, ≥100), stratified by institution.
Blinding	Because the patients were allocated to either the conventional treatment group or the app use group, it was clear which group they were allocated to and blinds were not possible.

Reporting for specific materials, systems and methods

We require information from authors about some types of materials, experimental systems and methods used in many studies. Here, indicate whether each material, system or method listed is relevant to your study. If you are not sure if a list item applies to your research, read the appropriate section before selecting a response.

Materials & experimental systems

n/a	Involved in the study	
☒	☐	Antibodies
☒	☐	Eukaryotic cell lines
☒	☐	Palaeontology and archaeology
☒	☐	Animals and other organisms
☐	☒	Clinical data
☒	☐	Dual use research of concern
☒	☐	Plants

Methods

n/a	Involved in the study	
☒	☐	ChIP-seq
☒	☐	Flow cytometry
☒	☐	MRI-based neuroimaging

Clinical data

Policy information about clinical studies

All manuscripts should comply with the ICMJE guidelines for publication of clinical research and a completed CONSORT checklist must be included with all submissions.

Clinical trial registration	We registered the study in the University Hospital Medical Information Network Clinical Trial Register (UMIN000033261).
Study protocol	Protocol papers have been published.<br>Kawai Y, Sankoda A, Waki K, et al. Efficacy of the Self-management Support System DialBetesPlus for Diabetic Kidney Disease: Protocol for a Randomized Controlled Trial. JMIR Res Protoc. 2021;10(8):e31061.
Data collection	This study was conducted in Japan. The recruitment period was from July 1, 2018 to August 31, 2019 and the study was completed in April 6, 2021.
Outcomes	The primary outcome was change after 12 months in UACR. We used logarithmically transformed data (log-UACR) due to a skewed distribution. In the primary analysis, the change in UACR was compared between groups by t-test.
